# Functional Characterization of the Lysine-Specific Histone Demethylases Family in Soybean

**DOI:** 10.3390/plants11111398

**Published:** 2022-05-25

**Authors:** Mengshi Liu, Jiacan Jiang, Yapeng Han, Mengying Shi, Xianli Li, Yingxiang Wang, Zhicheng Dong, Cunyi Yang

**Affiliations:** 1 Guangdong Provincial Key Laboratory of Plant Molecular Breeding, Guangdong Subcenter of National Center for Soybean Improvement, College of Agriculture, South China Agricultural University, Guangzhou 510642, China; liumengshi@stu.scau.edu.cn (M.L.); volcano-jiang@stu.scau.edu.cn (J.J.); mingyingshi99@163.com (M.S.); 20203137032@stu.scau.edu.cn (X.L.); 2 State Key Laboratory of Genetic Engineering and Institute of Genetics, Institute of Plant Biology, School of Life Sciences, Fudan University, Shanghai 200240, China; hanyapeng127@163.com (Y.H.); yx_wang@fudan.edu.cn (Y.W.); 3 Guangdong Laboratory of Lingnan Modern Agriculture, Guangzhou 510642, China; 4 College of Life Science, South China Agricultural University, Guangzhou 510642, China; 5 Guangzhou Key Laboratory of Crop Gene Editing, Innovative Center of Molecular Genetics and Evolution, School of Life Sciences, Guangzhou University, Guangzhou 510006, China

**Keywords:** soybean, *LSD* gene family, H3K4 demethylase

## Abstract

Histone modifications, such as methylation and demethylation, have crucial roles in regulating chromatin structure and gene expression. Lysine-specific histone demethylases (LSDs) belong to the amine oxidase family, which is an important family of histone lysine demethylases (KDMs), and functions in maintaining homeostasis of histone methylation. Here, we identified six *LSD-like* (*LDL*) genes from the important leguminous soybean. Phylogenetic analyses divided the six GmLDLs into four clusters with two highly conserved SWRIM and amine oxidase domains. Indeed, demethylase activity assay using recombinant GmLDL proteins in vitro demonstrated that GmLDLs have demethylase activity toward mono- and dimethylated Lys4 but not trimethylated histone 3, similar to their orthologs previously reported in animals. Using real-time PCR experiments in combination with public transcriptome data, we found that these six *GmLDL* genes exhibit comparable expressions in multiple tissues or in response to different abiotic stresses. Moreover, our genetic variation investigation of *GmLDL* genes among 761 resequenced soybean accessions indicates that *GmLDLs* are well conserved during soybean domestication and improvement. Taken together, these findings demonstrate that GmFLD, GmLDL1a, and GmLDL1b are bona fide H3K4 demethylases towards H4K4me1/2 and GmLDLs exist in various members with likely conserved and divergent roles in soybeans.

## 1. Introduction

In eukaryotes, the histones wind DNA into a nucleosome, and then the DNA–protein complexes are further compressed to form a complex structure known as chromatin. The core nucleosome consists of two copies of H2A, H2B, H3, and H4, and its tails are accessible for post-translational modifications (PTMs), including phosphorylation, ubiquitination, acetylation, methylation glycosylation, ADP-ribosylation, and SUMOylation; these modifications have essential roles in transcriptional regulation and chromatin architecture [1,2]. Histone methylation is a complex post-translational modification, which occurs at lysine or arginine residues and distinct positions by adding different numbers of methyl groups. Histone methylation can either be an active or repressive mark, depending on the location and degree of methylation, for example, tri-methylation of histone H3 at lysine 4 (H3K4me3) is generally considered an active mark for transcription, whereas dimethylation of histone H3 at lysine 9 (H3K9me2) is linked to transcriptional repression [3]. Methylation of H3K4 is an important histone mark in plants, including mono-, di-, and trimethylation modifications (H3K4me1, H3K4me2, and H3K4me3, respectively), which are written by different classes of SET domain-containing methyltransferases and erased by histone demethylases [4,5]. To date, histone demethylases have identified two classes, the Jumonji C (JmjC) domain-containing proteins and the amine oxidase domain family (LSD), with distinct enzymatic mechanisms and substrate specificity [6,7]. The JmjC domain-containing proteins belong to the dioxygenase superfamily and are able to remove methyl groups from methylated lysine residues using Fe^2+^ and α-ketoglutarate as cofactors [8,9]. LSD proteins use the flavin adenine dinucleotide (FAD) as a cofactor to remove both mono- and dimethylation of histone H3 at the lysine tail [6,10].

Human lysine-specific demethylase 1 (HsLSD1, also known as KDM1A) was the first identified histone demethylase, and it belongs to the flavin adenine dinucleotide-dependent amine oxidase superfamily [6]. HsLSD1 contains three main domains: an N-terminal SWIRM domain, a C-terminal amine oxidase-like domain, and a protruding Tower domain [11]. The SWIRM domain is critical for the protein–protein interactions and contributes to the steadiness of the protein. The amine oxidase-like domain was defined as the catalytic center in regulating the enzymatic activity responsible for targeting substrate proteins [12,13]. The Tower domain acts as a hub for the interaction with other proteins, especially molecular adaptors, such as the corepressor protein [14]. LSD1 has a homolog called LSD2 (also known as KDM1B), which bears a catalytic amine oxidase domain and a SWIRM domain. However, unlike LSD1, LSD2 does not contain a Tower domain but comprises an N-terminal zinc-finger domain [15,16], indicating that the interaction proteins and transcriptional regulatory network of LSD2 are different from those of LSD1.

In *Arabidopsis thaliana*, four LSD1 homologs have been identified: LSD-like 1 (LDL1), LDL2, LDL3, and FLOWERING LOCUS D (FLD), all bearing an amine oxidase-like domain and a SWIRM domain [17,18]. Similar to HsLSD1, *Arabidopsis* LDL/FLDs can specifically demethylate H3K4me1 and H3K4me2 peptides. In *ldl1*, *ldl1ldl2*, and *fld* mutants, the H3K4 methylation level of target genes is increased, supporting the H3K4 demethylase activity of LDL1, LDL2, and FLD [17]. Previous studies showed that *Arabidopsis* LDL/FLD plays a critical role in the control of flowering. *fld* and *ldl1ldl2* mutant plants show late-flowering phenotypes; consistently, H3K4me2 levels are increased in *fld* at *FLOWERING LOCUS C* (*FLC*, a MADS-box transcriptional regulator that inhibits floral transition) as compared to wild type plants. Consistently, *FLC* transcription is also downregulated by LDL1 and LDL2, which act in partial redundancy with FLD, the latter playing a more prominent role [17,19,20]. LDL1 and LDL2 also control the H3K4 methylation state of FLOWERING WAGENINGEN (FWA), a homeodomain-containing transcription factor in repressing plant flowering [17]. Unlike *fld* and *ldl1ldl2* mutants, the *ldl3* mutant displays an early-flowering phenotype, and the expression of *FLC* is downregulated, indicating that LDL3 has an opposing effect in comparison to the others [21]. Additionally, the function of *LDL/FLD* homologs in regulating the flowering gene expression through controlling the histone methylation level was also found in other plant species [22,23,24]. Other studies presented evidence that the *LDL/FLD* gene family is also involved in several development and stress defense processes, such as root elongation, seed dormancy, plant immune responses, and the circadian clock [25,26,27,28,29].

To date, our knowledge about the LSD family is limited to the *Arabidopsis*, and the functions of this family in the soybean, an important economic crop for plant proteins and oils, remain poorly understood. In this study, a total of six soybean *LSD-like* genes, *GmLDLs*, were identified and characterized molecularly. Recombinant GmLDLs were expressed and purified from a bacterial heterologous system and exhibited catalytic demethylase activity towards H4K4me1/2. Our results pave the way for further research on the potential function of LSD-like proteins in regulating gene expression as well as in soybean growth, development, and stress responses.

## 2. Results

### 2.1. Identification and Characterization of GmLDLs

**Figure 1:**
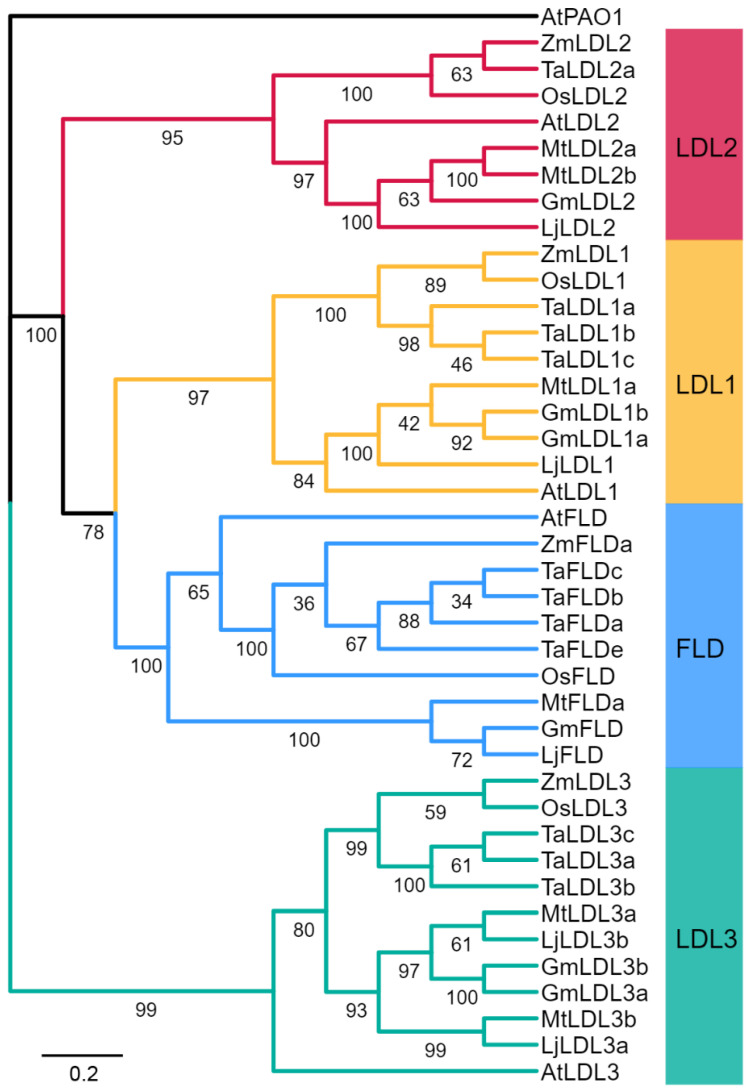
Phylogenetic relationships of the LSD-like genes (LDLs). Phylogenetic relationships of the LDLs from the soybean (Gm), *Arabidopsis* (At), *Oryza sativa* (Os), *Triticum aestivum* (Ta), *Zea mays* (Zm), *Medicaogo truncatula* (Mt), and *Lotus corniculatus* (Lj). The phylogenetic tree was constructed by the neighbor-joining method using MEGA 11.0. The bootstrap values were 1000 replications for major branches. LDL family genes were divided into four groups; branches are colored differently. The sequence of *AtPAO1* (At5G137900) was used as an outgroup.

To identify GmLDL genes, the Arabidopsis LDL family amino acid sequences were used to perform a genome-wide search in soybeans. A total of six GmLDL members with the typical flavin amine oxidase domain and SWRIM domain were identified (Figure S1); detailed information on these genes is listed in Table S1. The polypeptide sequences of GmLDLs ranged from 734 to 1907 amino acids (aa) in length, the molecular weight (MW) of 80.6 kDa (GmLDL1a) to 207.9 kDa (GmLDL3a), and their inferred isoelectric point (pI) varied from 5.45 to 6.09. To explore the classification and phylogenetic relationship of GmLDLs, we constructed a phylogenetic tree with the LDL family proteins from the soybean, *Arabidopsis*, *Oryza sativa*, *Triticum aestivum*, *Zea mays*, *Medicaogo truncatula*, and *Lotus corniculatus*. The results revealed that the six GmLDLs were classified into four clades based on the bootstrap values and phylogenetic topology (Figure 1), namely, FLD (GmFLD), LDL1 (GmLDL1a and GmLDL1b), LDL2 (GmLDL2), and LDL3 (GmLDL3a and GmLDL3b). In addition, compared with a previous study [22], we identified two additional LSD-like genes in the soybean. For convenience, we designated them as GmFLD, GmLDL2, GmLDL1a, GmLDL1b, GmLDL3a, and GmLDL3b based on their relationship to the AtLDL family (Figure 1).

Different combinations of exons and introns can lead to diverse gene functions [30,31]. To further explore the structural diversity of the *LSD-like* genes in the soybean, the characteristics of exon/intron structures were analyzed by the Gene Structure Display Server according to the genomic DNA annotations. As shown in Figure S2B, different subfamilies displayed variations in exon/intron structures; for example, *GmLDL1b* did not contain any introns, *GmLDL1a* and *GmLDL2* contained only one intron, while the remaining genes had more than two introns. Moreover, combined with the result of phylogenetic trees, we found that the group of LDL1 (*AtLDL1* and *GmLDL1a*, *GmLDL1b*) comprised one or no introns and the LDL3 group (*AtLDL3* and *GmLDL3a*, *GmLDL3b*) possessed seven introns, which indicates that exon/intron patterns within the same phylogenetic classification group showed great similarity.

**Figure 2:**
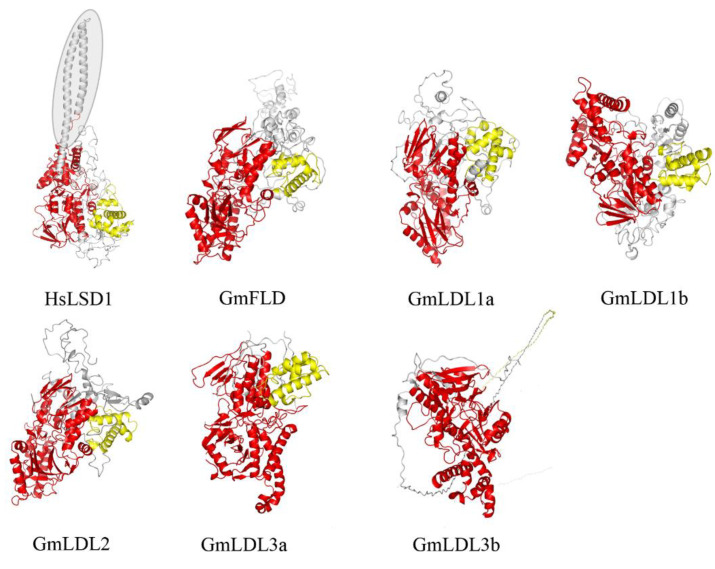
Three-dimensional structures of HsLSD1 and GmLDLs. The three-dimensional structure of human LSD1 (HsLSD1) is determined by Forneris et al. [32] in complex (substrate-like) peptide inhibitors. The three-dimensional structures of GmLDLs were obtained through molecular modeling approaches using the intensive mode. The conserved domains, SWRIM and amine oxidase, are represented in yellow and red, respectively. The shade indicates the Tower domain. The GmLDL3a and GmLDL3b just show the structures of conserved domains.

To further characterize soybean LDL homologs, we built their three-dimensional structure models through Phyre 2 using the intensive mode. Unlike the HsLSD1, the GmLDLs do not have the protruding Tower domain (Figure 2), which is consistent with AtFLD, AtLDL1, and AtLDL2 [18]. The models of GmLDLs show that the three-dimensional structure of SWRIM and the amine oxidase domain were similar to HsLSD1, except for GmLDL3b, whose SWRIM domain did not form the α helix structure, such as HsLSD1 (Figure 2). The previous study indicated that despite the lack of a Tower domain, the AtLDL1 and mouse LSD2 can remove the methyl group of H3K4 [18], which suggests that the members of the soybean LSD-like family may have demethylase activity, such as AtLDL1.

### 2.2. Expression Profile of GmLDLs

**Figure 3:**
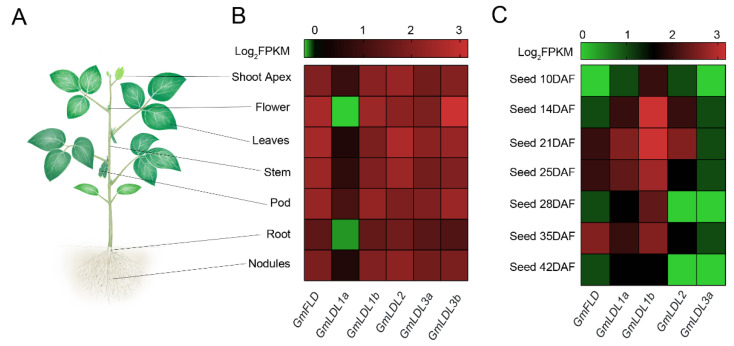
Expression patterns of *GmLDL* genes. (**A**) A diagram of the investigated plant tissue. These tissues include shoot apex, flower, leaves, stem, pod, root, and nodules. (**B**) Expression patterns of *GmLDL* genes in different tissues. (**C**) Expression patterns of *GmLDL* genes at different developmental stages of the seed. The color scale above the heat map indicates the gene expression levels. The green color indicates a low expression level and the red color indicates a high expression level, the number 3 is the highest, and 0 is the lowest.

Gene expression patterns refer to potential biological function. We explored spatiotemporal expression patterns of *GmLDLs* using publicly available RNA-seq data in soybeans. The results revealed that the expression levels and patterns of *GmLDLs* in different tissues varied considerably. The *GmFLD* showed higher expression levels in shoots compared to roots, especially in flowers and leaves. The *GmLDL1a* displayed extremely lower expression levels in all samples, while its paralog, *GmLDL1b*, was highly expressed in flowers and pods. *GmLDL2* had a high expression level in leaves, while the expression of *GmLDL3b* was strong in flowers (Figure 3B), and the qRT-PCR experiments confirmed the results (Figure S3). Taken together, the differential expression patterns of *GmLDL*s indicate that they may have multiple functions in soybean growth and development. 

To understand the potential functions of *GmLDL*s in seed development, the RNA-seq data were used to analyze their expression patterns (no information for *GmLDL3b*). Generally, some *GmLDL*s were broadly expressed at different developmental stages of soybean seeds (Figure 3C). The expression levels of some genes, however, were not only varied between tissues but also distinguished in different developmental stages. For instance, the highest expression of *GmLDL1b* was observed at the whole stage, except DAF (day after flowering) 42, indicating that it may play a general role in seed development. *GmFLD* exhibited preferential expression at a later stage, as the expression level was found to be high in DAF 35, implying the role in later seed development and ripening. In addition, *GmLDL2* and *GmLDL1a* seem to have the same expression patterns in seed development, as both exhibited preferential expression at DAF 21, indicating that *GmLDL2* and *GmLDL1a* may have redundant functions in seed development.

**Figure 4:**
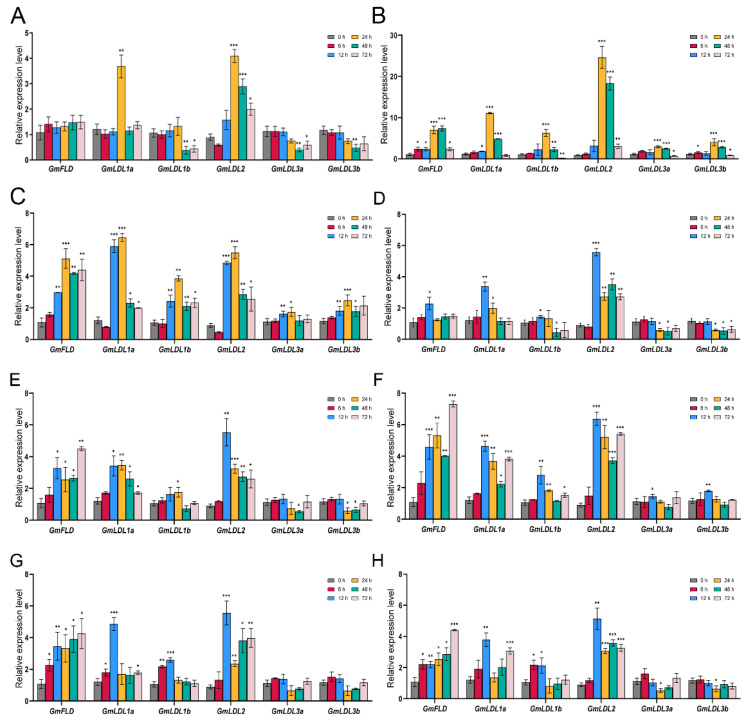
Expression patterns of *GmLDL* genes under different abiotic stresses. Two-week-old soybean seedlings were exposed to stress treatments as indicated below. Gene expression analysis was conducted by qRT-PCR using gene-specific primers. (**A**) Shade stress, (**B**) heat stress, (**C**) cold stress, (**D**) drought stress, (**E**) flooding stress, (**F**) salt stress, (**G**) low Pi stress, (**H**) low N stress. The mean values are from three independent biological replicates. Statistical analyses were performed by Student’s *t*-test (* *p* < 0.05, ** *p* < 0.01 and *** *p* < 0.001).

Plants are frequently responsive to both biotic and abiotic stresses, and they have evolved sophisticated adaptations and defense mechanisms [33]. In recent years, chromatin modifications, nucleosome positioning, and DNA methylation have been recognized as important components in such adaptations [34]. To investigate the potential roles of soybean *GmLDL* genes in response to various abiotic stress responses, we analyzed the conserved *cis*-elements in the promoter region (2 kb sequence upstream of the 5′UTR) of *GmLDL* genes using PlantCARE. The results show that *GmLDL* family numbers possessed a variety of *cis*-elements with putative responses to abiotic stress, hormone, and light-related (Figure S4). To further confirm how *GmLDL* genes responded to different abiotic stresses, we detected their expression levels under shade, heat, cold, drought, flooding, salt, and low Pi/N stress conditions by using qRT-PCR. Overall, some *GmLDLs* were significantly induced or repressed by multiple treatments (Figure 4). Under the shade condition, *GmLDL1a* and *GmLDL2* were significantly induced at 24 h compared with 0 h, while *GmLDL1b*, *GmLDL3a*, and *GmLDL3b* showed downregulated expression after 48 h. For heat and cold treatments, all members of the *GmLDL* family were upregulated from 6 h (heat stress) or 12 h (cold stress) to 48 h, with peak expression at 24 h. Under drought or flooding stress, the six *GmLDL* genes could be divided into two groups of different expression patterns: *GmLDL3a* and *GmLDL3b* decreased at a later time point (after 24 h), while the other *GmLDLs* were obviously induced at several time points. In addition, we observed that *GmLDLs* displayed different roles in salt, low Pi, or low N stress; *GmLDL3a* and *GmLDL3b* showed no obvious response to those stresses, while other genes were significantly upregulated during the NaCl, low Pi, or low N treatment.

### 2.3. The Genetic Diversity of GmLDLs in Resequenced Soybean Accessions

To explore the potential domestication selection of *GmLDLs*, we surveyed allelic variations in resequenced soybean accessions, including 95 wild soybeans (*Glycine soja*), 181 landraces, and 485 improved cultivars [35,36,37]. Generally, we did not find the SNPs or InDels in *GmFLD*, indicating that *GmFLD* is more conserved compared with other homologs. Furthermore, *GmLDL1a* had the largest mean number (2.72 per kb) of non-synonymous SNPs and InDels per kb sequence among these genes. It was noteworthy that only a few non-synonymous SNPs and InDels were found at the conserved sites, although some SNPs and InDels exist in *GmLDL* genes (Table 1).

The identification of genes associated with domestication and improvement is important for breeding superior varieties [35]. To detect the potential selective signals during the processes of soybean domestication (wild soybeans vs. landraces) and improvement (landraces vs. improved landraces), we compared the SNP and InDel distribution status of *GmLDLs* in 761 resequenced soybeans. As a result, a total of three domestication-selective sites in *GmLDL1a*, *GmLDL1b,* and *GmLDL2* were identified; however, none of these selective sites occurred at conserved domains (Table S2). Among them, the SNP Chr06:42304028 in *GmLDL2* (G-T, T corresponding to the reference genome Wm82.a2.v2) was strongly selected compared with others, as the distribution frequency of WT alleles ranged from 26.84% (*Glycine soja*) to 90.31% (improved cultivar, Table S2), indicating that *GmLDL2* may undergo selection during domestication and improvement or closely link to a selected site; alternatively, this could be a random result caused by genetic drift.

### 2.4. Heterologous Expression and Enzymatic Assay of GmLDLs

**Figure 5:**
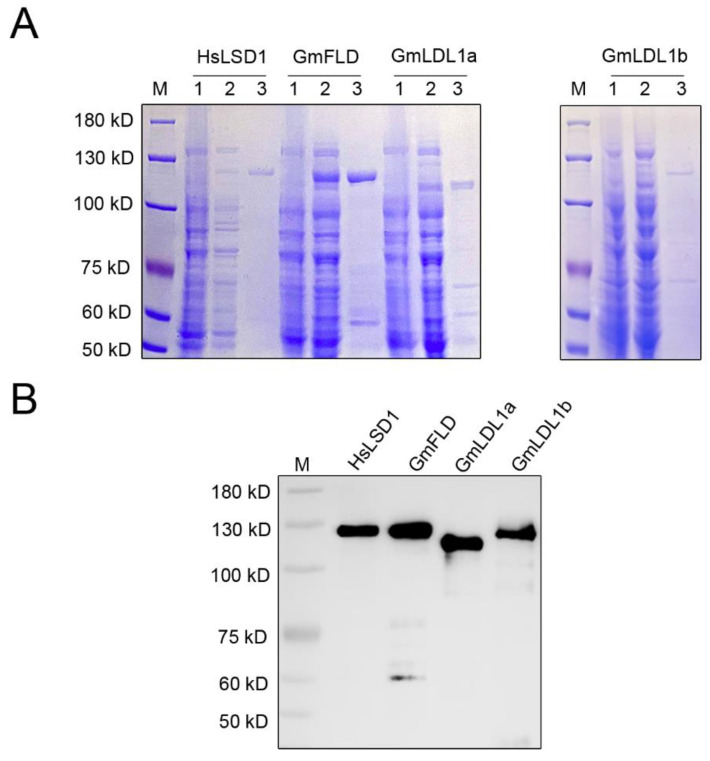
Purification of recombinant GmLDLs expressed in bacteria. (**A**) A SDS-PAGE analysis of the purified recombinant proteins. M, molecular mass marker; 1, total protein of *E. coli* cell before induction; 2, crude *E. coli* cell extracts; 3, pooled elution fractions from Glutathione Sepharose 4B resin. (**B**) Immunoblotting of the purified recombinant proteins using an α-GST antibody.

To investigate whether the GmLDL proteins are bona fide histone demethylase, we cloned the full-length CDS of *GmFLD*, *GmLDL1a,* and *GmLDL1b* into the vector pGEX 6p-1, infused with a GST tag on the N-terminal region of each protein (named GST-GmFLD, GST-GmLDL1a, and GST-GmLDL1b, respectively). Additionally, *HsLSD1* was cloned into the vector pGEX 6p-1 (GST-HsLSD1) as a control. The GST fusion proteins were expressed in *E. coli* cells and affinity-purified. The purified proteins were then analyzed by SDS-PAGE. The results showed that the purified proteins displayed an apparent molecular mass between 100 and 130 kDa (Figure 5A), the molecular mass expected for the recombinant proteins from the amino acid sequence analysis together with the GST. Furthermore, an immunoblotting analysis using an anti-GST tag antibody confirmed recombinant protein accumulation in the soluble bacterial extracts (Figure 5B).

**Figure 6:**
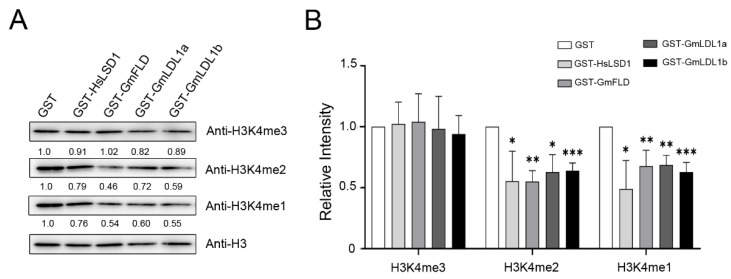
GST-GmLDLs exhibit H3K4me1/2 demethylase activity in vitro. (**A**) *E. coli*-expressed and affinity-purified proteins were incubated with calf thymus histone, and the H3K4 methylation status was determined using methylation-specific antibodies. GST-HsLSD1 was used as a positive control and anti-H3 was used as a loading control. (**B**) Quantification analysis of (**A**) by ImageJ 1.8.0. The mean values are from three independent biological replicates. Statistical analyses were performed by Student’s *t*-test (* *p* < 0.05, ** *p* < 0.01 and *** *p* < 0.001).

To examine the histone demethylase activity of GmLDLs, we incubated GST-tagged GmLDL with calf histone, and the methylation status was determined using H3K4me1/me2/me3-specific antibodies. Immunoblotting analyses indicated that GmFLD, GmLDL1a, and GmLDL1b are able to demethylate H3K4me2 and H3K4me1 peptides (Figure 6), similarly to HsLSD1. In combination with the other two independent experiments (Figure S5), we found that specific activity with the H3K4me2 peptide of those recombinant proteins is higher than that with the H3K4me1 peptide. Furthermore, the results show that recombinant proteins are not able to demethylate H3K4me3. The significant reduction in the methylation signal on K4 in the presence of recombinant proteins was not due to the degradation of the H3K4 peptides since those the H3 peptides remained unchanged before and after incubating with these demethylases. As a control, GST failed to catalyze the same enzymatic reaction (Figure 6 and Figure S5). Moreover, we found that recombinant proteins were not able to demethylate H3K9me2, H3K27me3, and H3K36me3 (Figure S6). Taken together, these findings demonstrate that GmFLD, GmLDL1a, and GmLDL1b are H3K4 demethylases with apparent H3K4me1/2 demethylation activity.

## 3. Discussion

The methylation of H3K4 is an important indicator of gene transcription. As a member of demethylases, LSD can regulate gene expression by modulating the H3K4 methylation level of target genes. Although the regulatory mechanisms of the *LSD*-*like* family genes are well studied in *Arabidopsis*, the roles of this family are rarely reported in the soybean. Hence, our study conducted a foundational exploration of the soybean *LSD-like* family, including gene structure, phylogenetic relationships, three-dimensional structure, expression pattern, genetic diversity, and histone demethylase activity.

The soybean is a paleopolyploid plant that has experienced at least two rounds of whole-genome duplication (WGD) events, resulting in a highly duplicated genome with nearly 75% of the genes present in multiple copies [38]. The phylogenetic analysis of the *LSD-like* gene family in *Arabidopsis* and soybean indicated *AtLDL1* and *AtLDL3* have two orthologs in the soybean genome, while *AtFLD* and *AtLDL2* only have a single ortholog in the soybean genome (Figure 1). We speculate that another paralogue gene may have been lost during the evolution of the soybean. Data from amino acid sequence and three-dimensional structure analyses suggest that GmLDLs, similar to their homolog in *Arabidopsis*, have two highly-conserved SWRIM and amine oxidase domains, but lack the HsLSD1 Tower domain (Figure 1) [18,21]. Moreover, the expression patterns of different *GmLDLs* seem to be distinct from the homologs of *Arabidopsis*. For example, the *Arabidopsis FLD* and *LDL2* are preferentially expressed in the shoot apex [17,21], while the expression levels of both *GmFLD* and *GmLDL2* are relatively higher in the leaves (Figure 3B), suggesting that the functions of *GmFLD* and *GmLDL2* may differ from their homologs *AtFLD* and *AtLDL2*.

Histone demethylases as chromatin modifiers, play significant roles in plant responses to various stresses [39,40]. Several studies indicate that histone methylation contributes to the regulation of gene expression under different abiotic stresses in soybeans, especially salt stress, finding that the activation or repression of salt-inducible genes is correlated with histone methylation modifications under salinity conditions [41,42,43,44,45]. To further investigate the putative roles of *GmLDLs* in soybean responses to abiotic stresses, we examined the expression patterns under shade, heat, cold, drought, flooding, salt, low Pi, and low N conditions using qRT-PCR. Our results showed that *GmLDLs* are significantly induced or repressed by multiple treatments, which agree with the findings of several putative *cis*-elements related to abiotic stress and light responsiveness (Figure 4 and Figure S4). For example, the light-responsive elements were found in the promoters of all *GmLDLs*, and their expression levels were either upregulated or repressed under shade conditions except *GmFLD*. The promoters of *GmFLD*, *GmLDL1b,* and *GmLDL3a* contain the drought-related MBS *cis*-element, while they exhibit different regulation models after drought treatment. The transcription of *GmFLD* and *GmLDL1b* was obviously increased against drought, whereas the expression of *GmLDL3a* slightly decreased compared with the control, which indicates that *GmFLD*/*GmLDL1b* and *GmLDL3a* may have antagonistic responses to drought stress. We speculate that GmLDLs act as chromatin remodeling factors of stress-inducible genes to regulate their expressions in response to different abiotic stresses.

The detection of genome-wide genetic diversity and the identification of genes relevant to domestication and improvement will be helpful for future crop improvement [35,46]. In this study, we investigated the allelic variations of *GmLDLs* in 761 resequenced soybean accessions. Our data reveal that these genes are well conserved, especially *GmFLD* since no non-synonymous SNPs and InDels were discovered at the conserved sites (Table 1). By comparing the previous study, which identified 121 domestication-selective sweeps, 109 improvement-selective sweeps, and 140 selective sweeps [35,36], we found that none of these genes exist in those selective sweeps (Table S2). Additionally, although we identified three domestication-selective non-synonymous SNPs in *GmLDLs*, they did not occur at the conserved sites (Table S2). These results show that the *GmLDL* genes may not undergo selection during domestication and improvement. It was noteworthy that the missense SNPs at the conserved SWRIM and amine oxidase domains—especially the amine oxidase domain, which is the catalytic center to regulate the enzymatic activity—may occur at the substrate-binding residues and lead to the change in protein function. Comparing the biochemical properties of the two proteins will be helpful to uncover the functional significance of those missense mutations.

The enzymatic assay shows that GmFLD, GmLDL1a, and GmLDL1b are lysine-specific histone demethylases with substrate specificity, such as HsLSD1. In particular, recombinant proteins are H3K4me1 and H3K4me2 demethylases, but not H3K4me3, H3K9me2, H3K27me3, and H3K36me3 demethylases, consistent with AtLDLs [18,21]. Based on the present results, we speculate that GmLDL2, GmLDL3a, and GmLDL3b can also remove the methyl group of H3K4me1 and H3K4me2, since they contain the complete conserved domain—SWRIM and amine oxidase—and the functional domain arranged in the same pattern as that in other GmLDLs (Figure 2). The data presented in this paper represent the first demonstration that the GmLDL family are lysine-specific demethylases in vitro. Previous studies have shown that *GmFLD* is a functional ortholog of the *Arabidopsis FLD*, which could complement the late-flowering phenotype of the *Arabidopsis fld* mutant plants by affecting the state of H3K4 methylation in FLC chromatin, whereas transgenic plants overexpressing *GmLDL2* did not show significant changes in flowering time, which may be explained by *AtLDL2*, which plays a minor and partially redundant role in controlling flowering of *Arabidopsi*s [21]. Another reasonable explanation is that the target genes of GmLDL2 may vary from AtLDL2. Although we demonstrated that the GmLDL family has the activity of histone demethylase in vitro, further characterization of the soybean LSD-like family remains to be performed using genetic and epigenetic approaches, such as loss-of-function and gain-of-function mutants, comparing the transcriptome and epigenome of mutants vs. wild type soybean plants, etc.

## 4. Materials and Methods

### 4.1. Identification of GmLDL Family Members

To identify *LSD-like* family members in soybeans, the sequence of *Arabidopsis* LDL proteins was used to search the Williams 82.a2.v2 reference genome in Phytozome 13 (available online: https://phytozome.jgi.doe.gov/pz/portal.html, accessed on 15 June 2021), with an expected value (e-value) cut-off 0.001. The genome sequences of *Arabidopsis* were retrieved from the TAIR database. The genome sequences of *Oryza sativa, Triticum aestivum, Zea mays, Medicaogo truncatula*, and *Lotus corniculatus* were identified from Phytozome 13. Then, the Pfam tool (available online: http://pfam.xfam.org/, accessed on 15 June 2021) was used to verify the retrieved GmLDL candidates with the typical conserved domains: SWIRM and amine oxidase.

### 4.2. Bioinformatics Analysis of LDL Family Genes

The full lengths of amino acid sequences of LDL members from *Arabidopsis* and soybean were aligned by ClustalX. Then, a phylogenetic tree was constructed using MEGA 11.0 with the neighbor-joining method and 1000 bootstraps. Figures representing the structural features were prepared with Evolview-v3 (available online: https://www.evolgenius.info/evolview-v3, accessed on 16 June 2021). The gene structures were analyzed by TBtools based on their genomic DNA annotations [47]. The physical and chemical parameters of GmLDLs were predicted by the ExPASy ProtParam tool, including amino acids, molecular weight, and *p*I (available online: https://www.expasy.org/, accessed on 16 June 2021). The *cis*-elements in each promoter (2 kb upstream of 5′UTR) of *GmLDL* genes were predicated using PlantCARE (available online: http://bioinformatics.psb.ugent.be/webtools/plantcare/html/, accessed on 16 June 2021).

### 4.3. Molecular Modeling

Molecular models of GmLDLs were built using the intensive mode of Phyre2 (available online: http://www.sbg.bio.ic.ac.uk/servers/phyre2/html/page.cgi?id=index, accessed on 26 November 2021), which can create a complete full-length model of a sequence through a combination of multiple template modeling and simplified ab initio folding simulations [48]. The best models were selected on the heuristics to maximize confidence, percentage identity, and alignment coverage. For the detection of sequence features, we used the Conserved Domain Database [49]. Figures representing the structural features were prepared with PyMol 2.5.2 (Schrodinger, New York, NY, USA).

### 4.4. Expression Patterns of GmLDL Family Genes

To examine the expression profiles of *GmLDL* family genes in different tissues and developmental stages of seeds, the transcriptome data of several different plant tissues and different developmental stages of seeds were downloaded from PPRD (available online: http://ipf.sustech.edu.cn/pub/plantrna/, accessed on 20 March 2022). The expression data were gene-wise normalized and the heatmap was drawn using Graphprism 9.0 (GraphPad Software, San Diego, CA, USA).

### 4.5. Genotyping the GmLDL Family

The single nucleotide polymorphisms (SNPs) and InDels of *GmLDLs* in soybean accessions were extracted from the released whole-genome resequencing data online (available online: http://47.96.185.131:70/iqgs/login, accessed on 10 September 2021). The genomic region was divided into the 5′-untranslated region (UTR), exon, intron, and 3′-UTR based on the genome annotation. The SNPs were classified as synonymous SNPs (no amino acid change), non-synonymous SNPs (cause amino acid substitutions), and stop-gain SNPs (generate a stop codon). InDels in the exonic regions were classified by whether they had frame-shift (3 bp insertion or deletion) mutations. SNPs and InDels with allele frequencies lower than 5% in the population were discarded.

### 4.6. Plant Materials and Treatments

The soybean seeds of Williams 82 were germinated in water at 25 °C under dark conditions and seedlings were subjected to a 12-h light and 12-h dark photoperiod and 30% humidity. Stress treatments were applied to two-week-old soybean seedlings. For shade stress, the soybean seedlings were exposed to 30% photosynthetically active radiation [50]; for heat stress, the soybean seedlings were exposed to 42 °C; for cold stress, the soybean seedlings were exposed to 4 °C; for drought stress, the soybean seedlings were treated with 15% PEG; for flooding stress, the soybean seedlings were treated with excess water that was maintained 5 cm above the sand; for salt stress, the soybean seedlings were treated with 150 mM NaCl; for low Pi stress, the soybean seedlings were treated with 5 μM KH_2_PO_4_; for low N stress, the soybean seedlings were treated with 1/4 N. After different time points, leaves were collected for RNA extraction. Total RNA was extracted from the soybean leaves (Williams 82) using the plant total RNA isolation kit (MAGE, Beijing, China), and the cDNA was synthesized by using the Prime Script RT reagent Kit with gDNA Eraser (Takara, Kusatsu, Japan). *GmACTIN3* was used as an internal control. The primers are listed in Supplementary Table S3. The three biological replicates were analyzed by *t*-test.

### 4.7. Gene Cloning and Plasmid Construction

Full-length CDSs of *GmFLD, GmLDL1a,* and *GmLDL1b* were amplified by RT-PCR from the cDNA sample and inserted into the vector pGEX 6p-1 at *BamH* I and *EcoR* I restriction sites to generate the GST-FLD, GST-LDL1a, and GST-LDL1b constructs. The gene primers are listed in Supplementary Table S3.

### 4.8. Recombinant Proteins Expression and Purification

The GST-HsLSD1, GST-FLD, GST-LDL1a, and GST-LDL1b plasmids were transformed into *E. coli* BL21 (DE3) cells. Expressions of recombinant proteins were induced by 0.2 mM isopropyl β-D-thiogalactoside (IPTG) at 16 °C for 20 h. Cells were resuspended in extraction buffer and disrupted by sonication. After centrifugation at 10,000× *g* for 30 min at 4 °C, the clear supernatant containing the soluble protein was further processed for recombinant protein purification with Glutathione Sepharose 4B resin (GE Healthcare, Merck, Darmstadt, Germany) according to the manufacturer’s manual. The recombinant proteins were analyzed by Western blot with anti-GST (Merck 71097, Darmstadt, Germany).

### 4.9. Histone Demethylation Assay

The in vitro demethylation assay was performed as previously described with minor modifications [51]. Briefly, a quantity of 7 μg of calf thymus histones (Sigma-Aldrich H9250, Shanghai, China) was incubated with affinity-purified GST-HsLSD1 (10 μg), GST-GmFLD (10 μg), GmLDL1a (10 μg), GmLDL1b (10 μg), or GST (10 μg) in 100 μL of reaction buffer (50 mM Tris, pH 8.5, 50 mM KCl, 5 mM MgCl_2_, 5% glycerol, and complete EDTA-free protease inhibitors) at 37 °C for 4 h. The reaction product was analyzed by immunoblotting with anti-H3K4me3 (Abcam ab8580, Cambridge, UK), anti-H3K4me2 (Millipore 07-030, Darmstadt, Germany), anti-H3K4me1 (Millipore 07-436, Darmstadt, Germany), anti-H3K9me2 (Abcam ab1220, Cambridge, UK), anti-H3K27me3 (Millipore 07-449, Darmstadt, Germany), anti-H3K36me3 (Abcam ab9050, Cambridge, UK), and anti-H3 (Abcam ab1791, Cambridge, UK). The relative intensity of the Western blot was quantified by ImageJ 1.8.0 (National Institutes of Health, Dickerson, FL, USA).

## 5. Conclusions

In conclusion, six *GmLDL* genes were identified in the soybean genome and they exhibited similar demethylase activity toward mono- and dimethylated Lys4 through recombinant GmLDLs in *E. coli*. We further found that *GmLDLs* are well conserved during soybean domestication and improvement, and qRT-PCR analysis showed that most *GmLDLs* responded to different abiotic stresses. Taken together, our results demonstrate that GmFLD, GmLDL1a, and GmLDL1b are bona fide H3K4 demethylases and may play an important role in gene expression in the soybean.

## Figures and Tables

**Table 1:** The SNP and InDel summary of *GmLDL* genes within sequenced soybean accessions.

Gene	Synonymous SNP	NS SNP	InDel	NS SNP and InDel/kb	NS SNP and InDel at CD
*GmFLD*	0	0	0	0	0
*GmLDL1a*	0	5	1	2.72	2
*GmLDL1b*	0	2	0	0.79	0
*GmLDL2*	0	2	0	0.90	0
*GmLDL3a*	0	4	0	0.71	1
*GmLDL3b*	0	2	0	0.35	1

Note: NS SNP: non-synonymous SNPs; NS SNP and InDel/kb: average number of no-synonymous SNPs and InDels per kb CDS sequence; CD: conserved domains.

## Data Availability

All data generated and analyzed in this study are included in this paper.

## Supplementary Materials

The following are available online at https://www.mdpi.com/article/10.3390/plants11111398/s1, Figure S1: Amino acid sequence alignment of HsLSD1 and GmLDLs. Numbers refer to amino acid residues, identical residues are shaded with black and similar residues are shaded with gray, the SWIRM domain is indicated with a black line, the amine oxidase domain is indicated with a red line, and the triangle indicates the spacer region of HsLSD1 omitted from the alignment; Figure S2: Gene structures of LDL genes of *Arabidopsis* (At) and soybean (Gm). (A) Phylogenetic relationships of LDL genes from *Arabidopsis* (At) and soybean (Gm), the phylogenetic tree was constructed by the neighbor-joining method using MEGA 11.0, the bootstrap values were 1000 replications for major branches. (B) Gene structures of LDL genes from *Arabidopsis* and soybean, UTR: untranslated regions, CDS: coding sequence; Figure S3: Expression of *GmLDLs* in different soybean tissues. The mean values are from three independent biological replicates; Figure S4: *Cis*-elements analysis in *GmLDL* gene family. (A) The different intensity colors and numbers of the grid indicate the numbers of different promoter elements in the *GmLDL* genes. (B) The position of elements involved in abiotic and biotic stress in each *GmLDL* gene promoter; Figure S5: The other two independent experiments of GST-GmLDLs demethylase activity in vitro; Figure S6: Western analyses of recombinant proteins activities on H3K9me2, H3K27me3, or H3K36me3. (A,B) *E. coli*-expressed and affinity-purified proteins were incubated with calf thymus histone, and the H3 methylation status was determined using methylation-specific antibodies. GST-HsLSD1 was used as a positive control and anti-H3 was used as a loading control. (C) Quantification analysis of (A,B) by ImageJ, the mean values are from two independent biological replicates; Table S1: The information about the *LDL* gene family in *Arabidopsis* and soybean. ‘+’ and ‘-’ indicate the genes are forward or reverse in the genome, MW is the protein molecular mass, *p*I is the theoretical isoelectric point; Table S2: The SNP distribution of *GmLDL* genes in resequenced 761 soybean accessions. The domesticated SNPs are shown in red; Table S3: The information of primers.
