# Supplementary figures and images for: Functional Characterization of the Lysine-Specific Histone Demethylases Family in Soybean
Source: Plants (Basel). 2022 May 25;11(11):1398. doi: 10.3390/plants11111398 (PMC9182794; doi:10.3390/plants11111398)

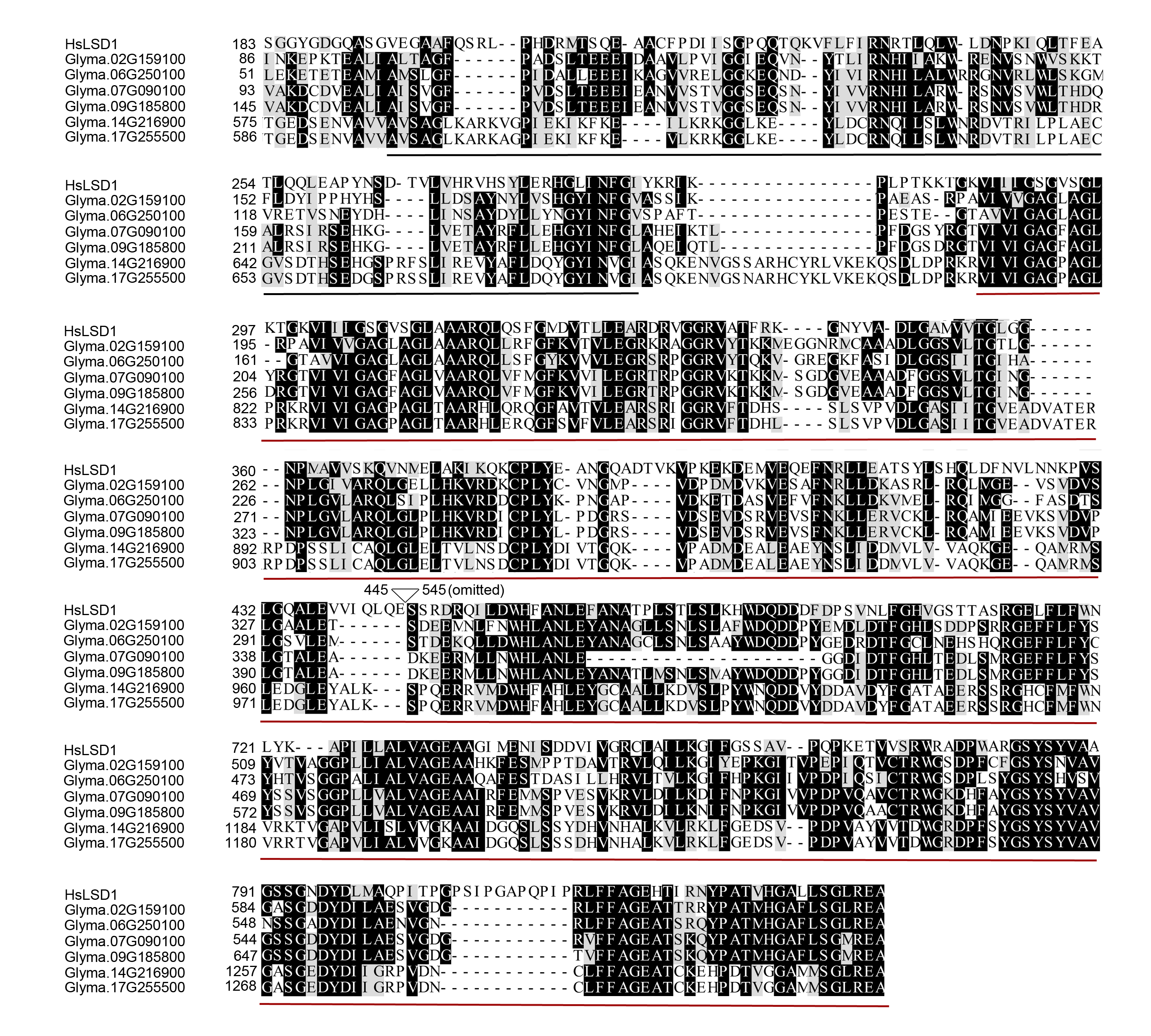

Supplement: Supplementary file 1 [file plants-11-01398-s001.zip › Figure S1.tif]

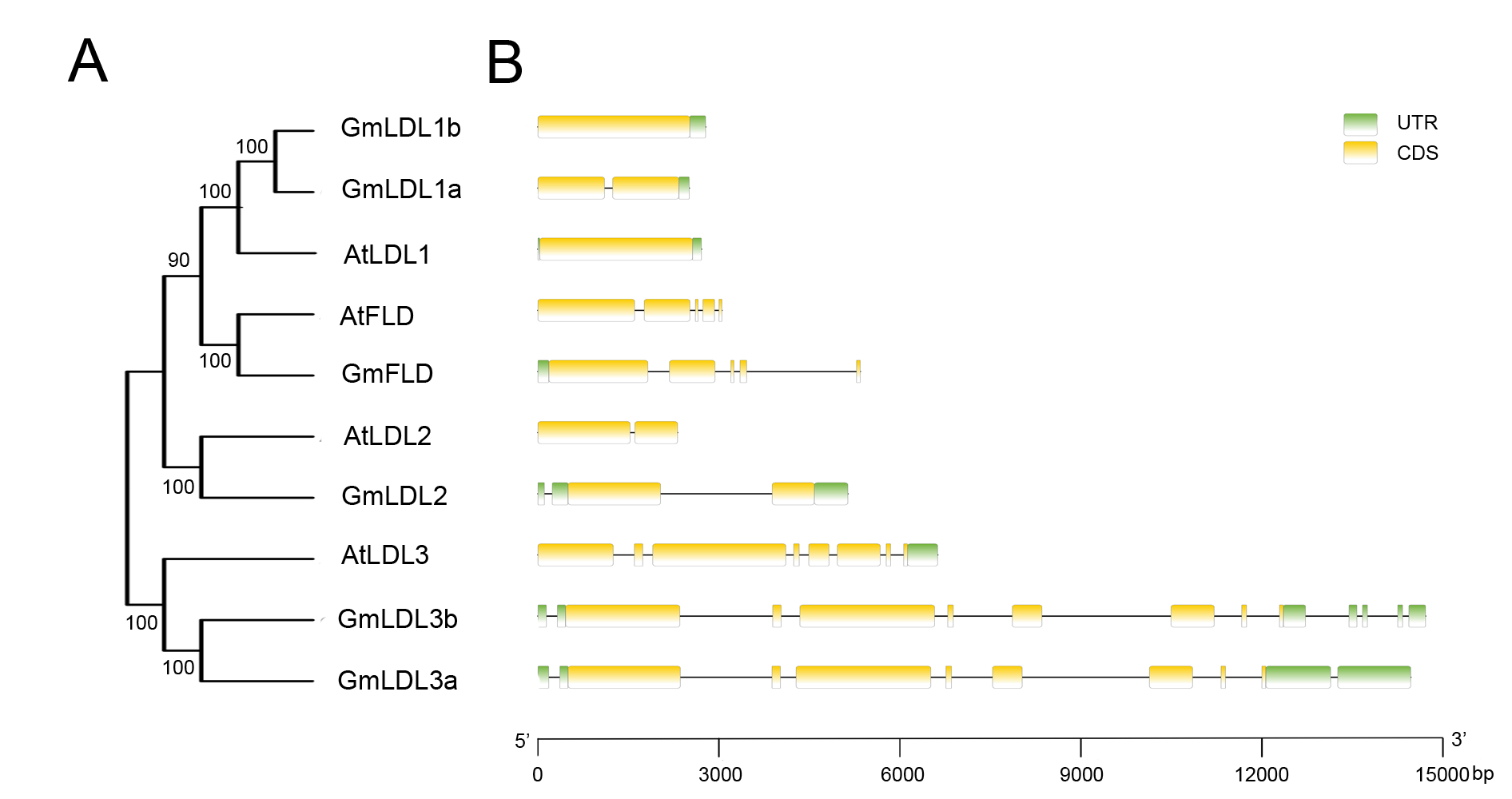

Supplement: Supplementary file 1 [file plants-11-01398-s001.zip › Figure S2.tif]

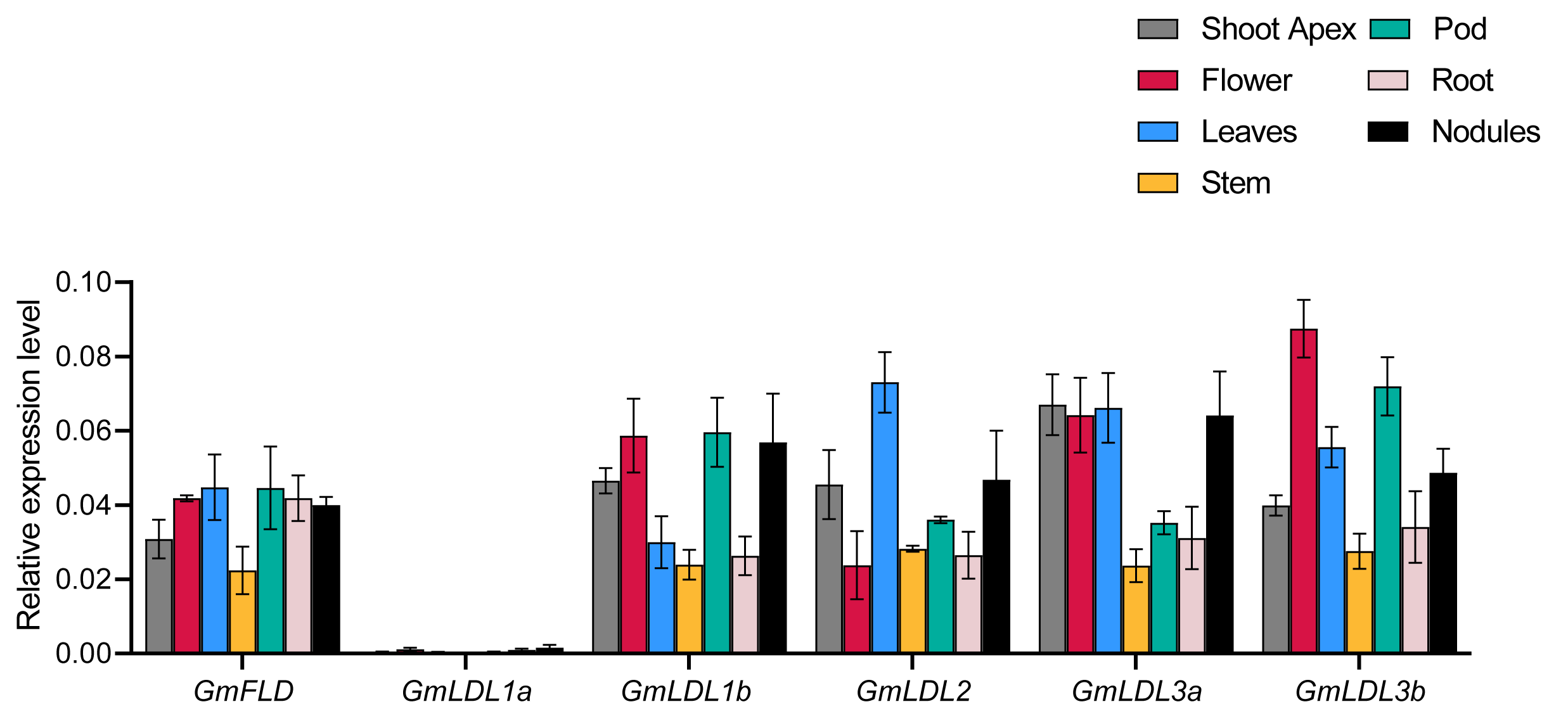

Supplement: Supplementary file 1 [file plants-11-01398-s001.zip › Figure S3.tif]

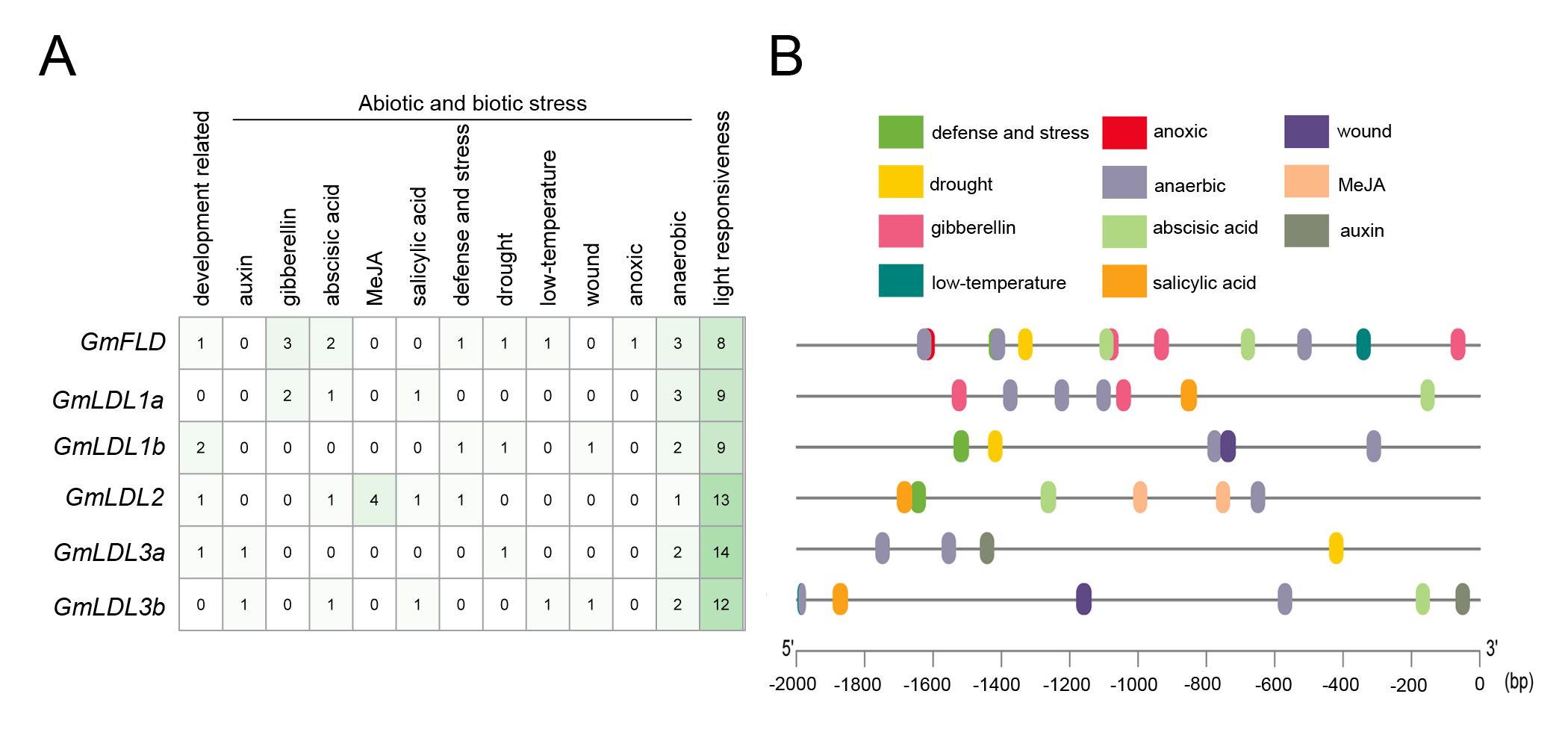

Supplement: Supplementary file 1 [file plants-11-01398-s001.zip › Figure S4.tif]

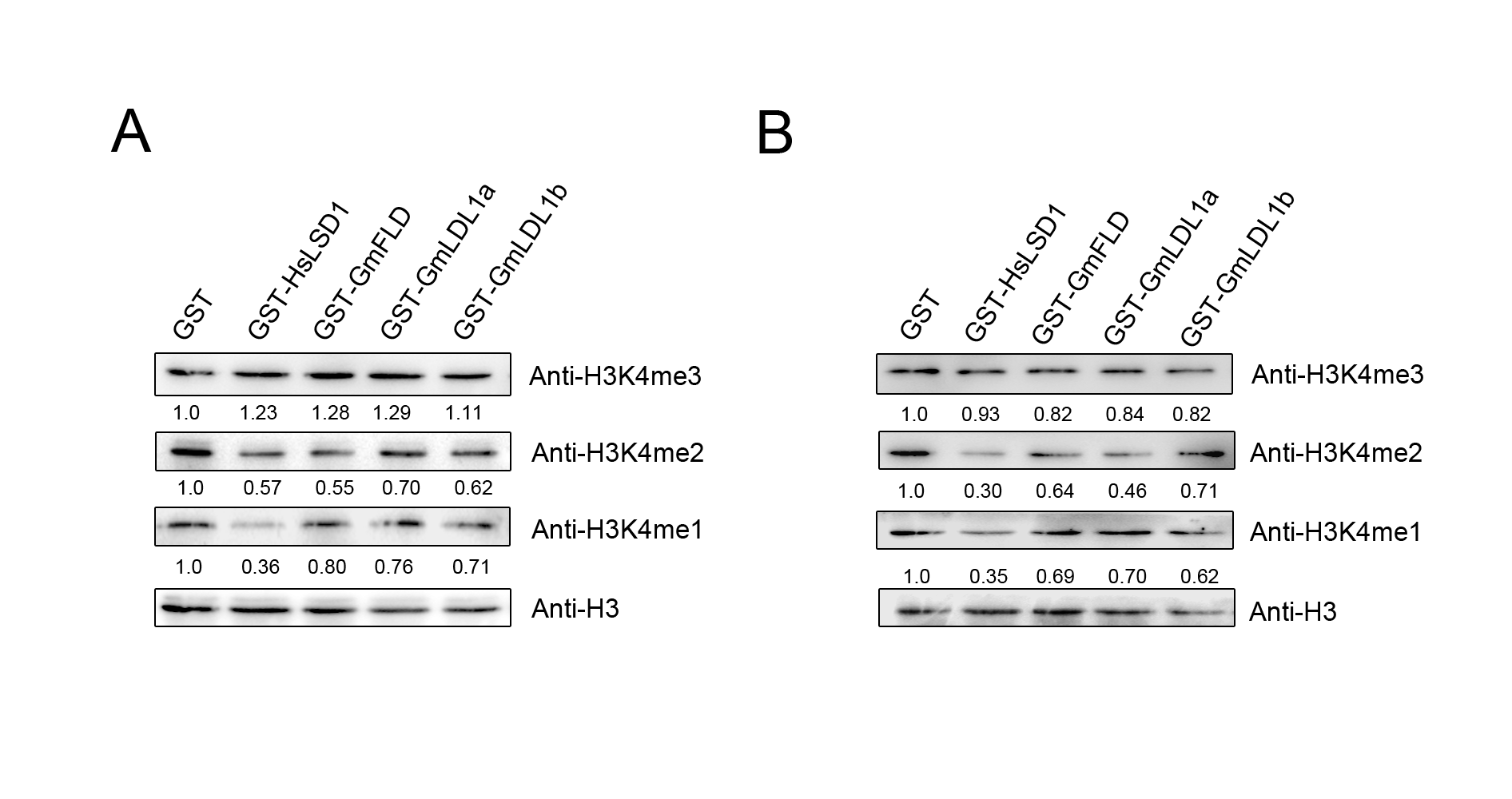

Supplement: Supplementary file 1 [file plants-11-01398-s001.zip › Figure S5.tif]

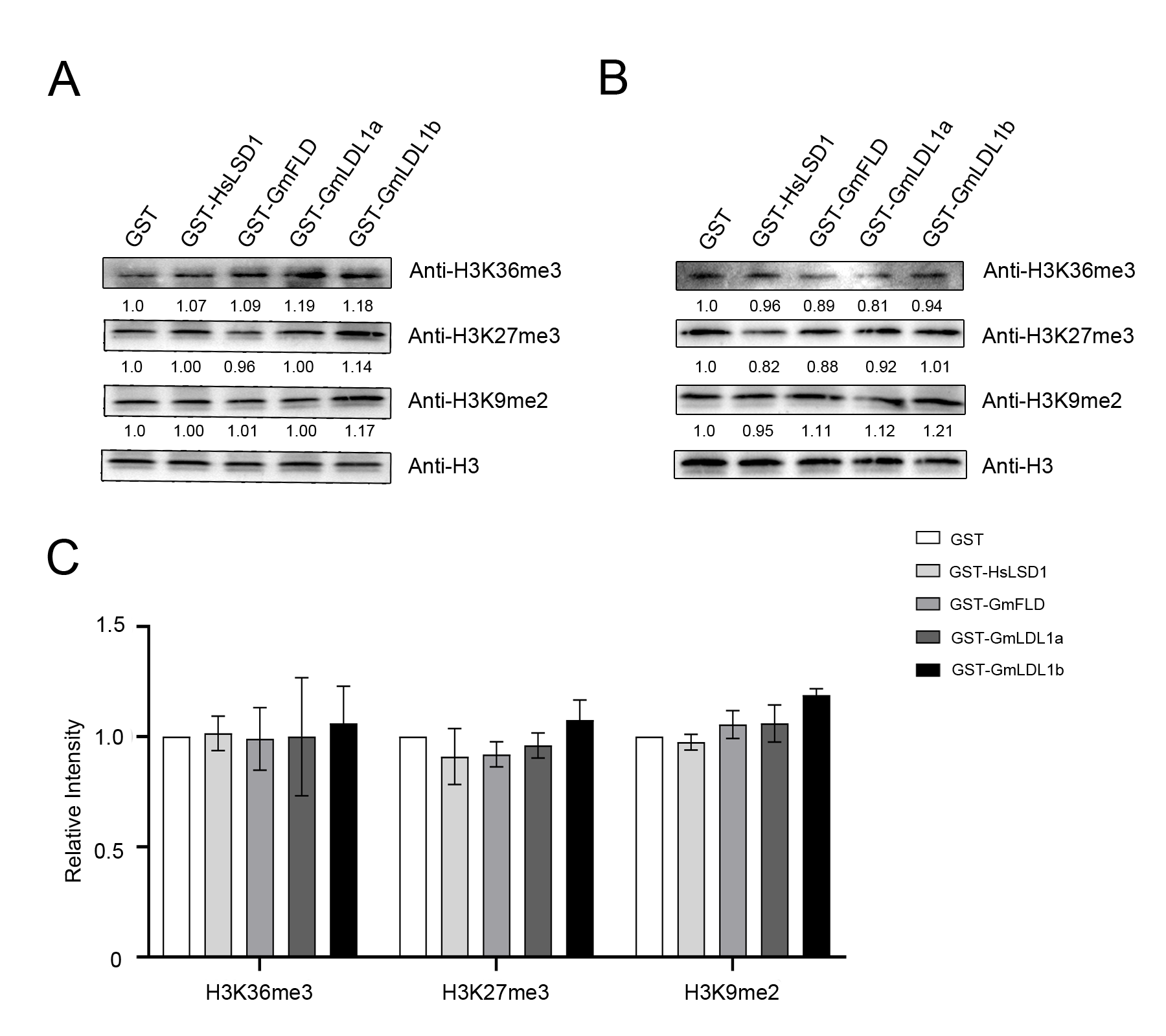

Supplement: Supplementary file 1 [file plants-11-01398-s001.zip › Figure S6.tif]

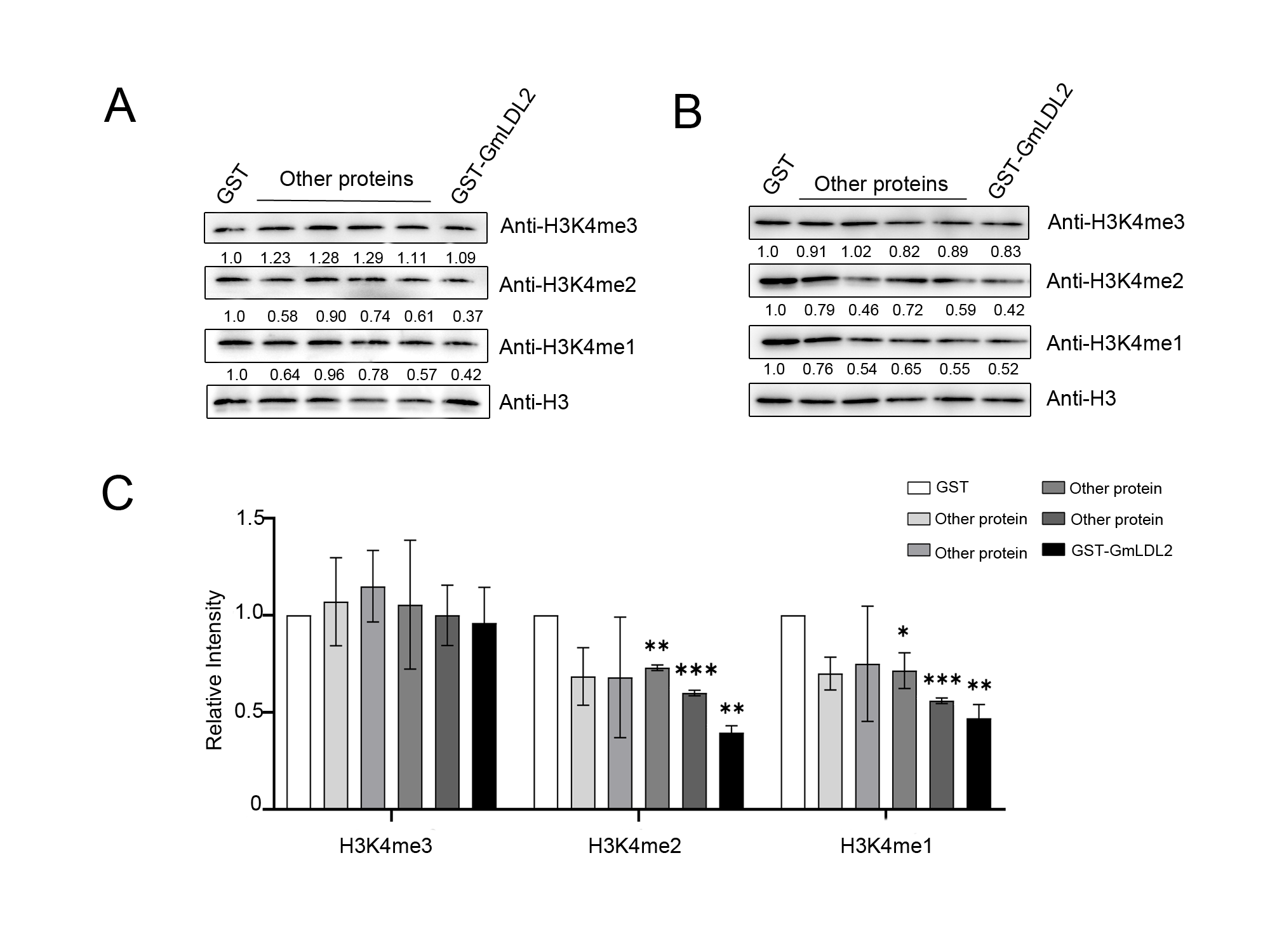

Supplement: Supplementary file 1 [file plants-11-01398-s001.zip › GmLDL2├╕╗ε╝∞▓Γ.tif]
